# Biofilm Formation and Degradation of Commercially Available Biodegradable Plastic Films by Bacterial Consortiums in Freshwater Environments

**DOI:** 10.1264/jsme2.ME18033

**Published:** 2018-09-29

**Authors:** Tomohiro Morohoshi, Taishiro Oi, Haruna Aiso, Tomohiro Suzuki, Tetsuo Okura, Shunsuke Sato

**Affiliations:** 1 Department of Material and Environmental Chemistry, Graduate School of Engineering, Utsunomiya University 7–1–2 Yoto, Utsunomiya, Tochigi 321–8585 Japan; 2 Center for Bioscience Research and Education, Utsunomiya University 350 Mine-machi, Utsunomiya, Tochigi 321–8505 Japan; 3 Process Development Research Laboratories, Plastics Molding and Processing Technology Development Group, Kaneka Corporation 5–1–1, Torikai-Nishi, Settsu, Osaka 556–0072 Japan; 4 Health Care Solutions Research Institute Biotechnology Development Laboratories, Kaneka Corporation 1–8 Miyamae-cho, Takasago-cho, Takasago, Hyogo 676–8688 Japan

**Keywords:** biodegradable plastic, poly(3-hydroxybutyrate-*co*-3-hydroxyhexanoate), biofilm, microbial community, degradation

## Abstract

We investigated biofilm formation on biodegradable plastics in freshwater samples. Poly(3-hydroxybutyrate-*co*-3-hydroxyhexanoate) (PHBH) was covered by a biofilm after an incubation in freshwater samples. A next generation sequencing analysis of the bacterial communities of biofilms that formed on PHBH films revealed the dominance of the order *Burkholderiales*. Furthermore, *Acidovorax* and *Undibacterium* were the predominant genera in most biofilms. Twenty-five out of 28 PHBH-degrading isolates were assigned to the genus *Acidovorax*, while the other three were assigned to the genera *Undibacterium* and *Chitinimonas*. These results demonstrated that the order *Burkholderiales* in biofilms functions as a degrader of PHBH films.

Marine pollution by smaller pieces of plastic, called micro-plastics, has become a serious global issue (2). Micro-plastics are defined as particles less than 5 mm in size with various chemicals and toxins attached to their surfaces (2). Microplastics are generated from marine plastic debris that is mainly fragmented by UV radiation in the marine environment. The use of biodegradable plastics is one solution to prevent the generation of microplastics in seawater. Biodegradable plastics are easily and rapidly broken down by various bacteria and fungi into CO_2_ and H_2_O (14). Various types of plastics have been evaluated for their degradability in soil environments. One of these biodegradable plastics, polyhydroxyalkanoate (PHA), is synthesized by a wide range of microorganisms and exhibits excellent biodegradability in soil environments (11). A biofilm is an assembly of surface-associated microbial cells that is enclosed in an extracellular polymeric substance matrix, such as polysaccharides (6). Biofilm formation by plastic-degrading soil isolates on plastic surfaces may be beneficial for the biodegradation of these complexes (7). Few studies have investigated the biodegradation of plastics through biofilm formation in aqueous environments. We previously demonstrated that one of the PHA derivatives, poly(3-hydroxybutyrate-*co*-3-hydroxyhexanoate) (PHBH), was degraded by the formation of a biofilm on its surface in seawater samples (9). Microplastic pollution has become a serious global issue not only in seawater, but also in freshwater environments (17). Although bacterial assemblage compositions on microplastic surfaces have been investigated in freshwater environments (17), the biodegradation of plastics by biofilm formation in freshwater environments has not yet been as extensively examined. Therefore, in the present study, we investigated biofilm formation and the biodegradation of general biodegradable plastic films in freshwater, and reported the composition of biofilm communities and isolation of bacteria with the ability to degrade biodegradable plastics.

Freshwater samples were collected at five locations in Japan in 2017 (Table 1). The biodegradability in freshwater of six types of general biodegradable plastic films: poly(lactic acid) (PLA), poly(butylene adipate-*co*-terephthalate) (PBAT), polybutylene succinate (PBS), poly(butylene succinate-*co-*butylene adipate) (PBSA), poly(ɛ-caprolactone) (PCL), and PHBH, was assessed (9). Biofilms were formed on plastic surfaces using a previously described method with slight modifications (9). Briefly, all plastic films had a thickness of approximately 100 μm, and were prepared by T die cast extrusion. Freshwater samples were mixed with 0.5 g L^−1^ NH_4_Cl as the N source and 0.1 g L^−1^ KH_2_PO_4_ as the P source. Rectangular plastic films with dimensions of 1×1.5 cm were cut from plastic sheets and soaked in 40 mL of freshwater samples in 50-mL vial bottles. Samples were incubated at 30°C with gentle shaking at 150 rpm until biofilms formed. After an incubation for 2 weeks, biofilm formation was observed on the surfaces of most plastic films and qualitatively evaluated from surface conditions (Table 1). PLA did not show any changes on its surface. The entire surfaces of the PHBH and PCL films were covered by biofilms in all of the freshwater samples tested. We previously demonstrated that biofilms were not detectable on the surfaces of PLA, PBAT, PBS, or PBSA films after an incubation in seawater samples (9). Biofilm formation and the biodegradation of plastics have been suggested to proceed more easily in freshwater environments than in seawater environments. In order to assess the structural conditions of the plastic films tested, biofilms were unglued from the surfaces of plastic films by vortexing and then air-dried. The transparencies of PCL and PHBH films were lost. A rugged surface and some holes were observed in PHBH films (Fig. 1). These results demonstrated that the bacterial community in the biofilm exhibited PHBH-degrading activity and caused the biodegradation of PHBH films.

Bacterial community structures were analyzed by 16S rRNA gene sequencing using MiSeq technology (Illumina, Hayward, CA, USA). Biofilms were unglued from the surfaces of plastic films by vortexing and collected by centrifugation at 10,000×*g* for 5 min. Total DNA was extracted from the collected biofilm samples by a DNeasy Blood & Tissue Kit (Qiagen, Tokyo, Japan) and diluted to 10 ng μL^−1^. The V1 and V3 regions of the 16S rRNA gene were amplified using V1 (27F) forward and V3 (534R) reverse primer pairs with added Illumina overhang sequences (18). Index tag sequences were added to the amplicons using a Nextera XT Index kit (Illumina) and then sequenced using a MiSeq Reagent Kit v3 (Illumina). The 301-bp paired-end reads were trimmed to remove low-quality ends (<15) and adapters using Trimmomatic software version 0.36 (3), and merged using fastq-join from the ea-utils toolkit with a minimum overlap of 10 bp and a maximum difference of 10% (1). The resultant high-quality reads were assembled using Geneious software version 9.1.8 and analyzed using the Ribosomal Database Project Database (RDP) Classifier in the Geneious 16S Biodiversity Tool (8).

The NGS analysis of the bacterial communities of biofilms that formed on PHBH films demonstrated the dominance of the class *Betaproteobacteria* in samples KS, KT, and YM (Fig. 2A). Sample KW contained a similar ratio of these two classes. The ratio of the class *Alphaproteobacteria* was slightly higher than that of the class *Betaproteobacteria* in biofilm sample KN, which was the only sample from a river. In comparisons with the bacterial community of the biofilm that formed on the PCL film, biofilm samples were obtained from PCL films incubated in freshwater samples from KS and KT. Consistent with the results obtained for PHBH films, the bacterial community of the PCL biofilm also showed the dominance of the class *Betaproteobacteria* (Fig. 2A). The order *Burkholderiales* was observed in all biofilm samples as the predominant order in the class *Betaproteobacteria*. A previous study reported that *Burkholderiales* isolated from soil exhibited the ability to degrade aliphatic biodegradable plastics (13). In the case of PHA degradation, bacteria belonging to *Burkholderiales* have been identified as one of the major PHA degraders in microbial communities in tropical soils (4). Therefore, the phylogenetic structure of the order *Burkholderiales* in all biofilm samples was elucidated. The results obtained showed that *Acidovorax* and *Undibacterium* were the predominant genera in most biofilms sampled from PHBH films (Fig. 2B). In sample KW, the genus *Pelomonas* was observed as the predominant genus and *Acidovorax*, *Undibacterium*, and *Ideonella* were the next dominant genera. The genera *Acidovorax*, *Pelomonas*, and *Ideonella* have been reported as PHA-accumulating organisms in full-scale waste-water treatment plants (12). PHA-accumulating bacteria are generally equipped with PHA-degrading enzymes, such as PHA depolymerase (PhaZ) (15). These findings suggest that PHBH films are degraded by PHA-degrading exoenzymes, which are secreted by the order *Burkholderiales* in freshwater as well as soil environments. We previously demonstrated that the family *Alteromonadaceae* was the most dominant bacteria in the biofilms that formed on PHBH films in seawater, whereas the order *Burkholderiales* was not detected (9). The difference in the dominant bacteria between seawater and freshwater samples appears to be due to the indigenous bacterial compositions in each sample.

In order to isolate PHBH-degrading bacteria from the bio-film that formed on the PHBH film, suspensions of biofilms were serially diluted with distilled water, spread on R2A (Difco, Tokyo, Japan) agar plates, and incubated at 30°C for 72 h. Colonies from each sample were randomly selected and transferred onto a fresh R2A agar plate. In order to detect PHBH-degrading activity, R2A agar medium containing 1 g L^−1^ PHBH powder (Kaneka Corporation, Hyogo, Japan) was prepared in the wells of a 24-well microplate. Bacterial colonies were inoculated on the center of R2A-PHBH agar medium. After an incubation at 30°C for 1 week, the development of clear zones around the colonies was evaluated as the degradation of PHBH. Five or six colonies each for all biofilm samples were randomly selected and their 16S rRNA genes were amplified from total DNA by PCR with Blend Taq-Plus DNA polymerase (Toyobo, Osaka, Japan) and the previously described primers, 27f (5′-AGAGTTTGATCMTGGCTCAG-3′) and 1525r (5′-AGGAGGTGWTCCARCC-3′) (10). PCR was performed using the following cycling parameters: 94°C for 30 s, 50°C for 30 s, and 74°C for 1 min for 27 cycles. Sequencing was performed using BigDye Terminator, ver. 3.1 and the Applied Biosystems 3500 Series Genetic Analyzer (Applied Biosystems, Foster city, CA, USA). The closest type-strain 16S rRNA gene relatives of each clone sequence were identified using the Ribosomal Database Project sequence match tool (5).

Regarding the results of the first screening for PHBH degradation, twenty-eight strains showed a clear zone on R2A-PHBH plates. The sequencing analysis of 16S rRNA genes revealed that most isolates (25 strains) were assigned to the genus *Acidovorax* (Fig. 3A). Based on the results of the phylogenetic analysis, 25 PHBH-degrading *Acidovorax* isolates were clearly divided into 3 groups at a similarity level of 99%. The 16S rRNA sequences of groups I, II, and III showed high similarity (more than 99% identity) to that of *Acidovorax facilis* CCUG 2113, *A. soli* BL21, and *A. delafieldii* ATCC 17505, respectively. In addition, each group contained *Acidovorax* strains isolated from at least 2 sampling sites in this study. In a previous study, the genus *Acidovorax* was isolated from soil as a degrader of the polymer of 3-hydroxybutyric acid (16). *Acidovorax* sp. DP5 produced the extracellular PHA depolymerase and degraded PHA films under alkaline conditions (15). A phylogenetic analysis revealed that DP5 belonged to group III with 99.6% identity. It was assumed that these three groups of PHBH-degrading *Acidovorax* strains were widespread in the various freshwater environments. Strains KW1 and YM2 showed high identity (more than 99% identity) to *Undibacterium pigrum* CCUG 49009. The nearest known type strain of strain KN3 was *Chitinimonas taiwanensis* LMG 22011 with 95.9% identity. All PHBHdegrading isolates belonged to the order *Burkholderiales* of the class *Betaproteobacteria*, which was the predominant order in the biofilm that formed on the PHBH film. *Acidovorax* and *Undibacterium* were the predominant genera in PHBH bio-film samples, as described above (Fig. 2B). In order to evaluate their abilities to biodegrade PHBH plastic films, the five strains selected from each PHBH-degrading bacterial group were cultured for 3 d on R2A-PHBH agar plates and R2A liquid medium containing 1×1.5-cm PHBH films. All of the tested isolates not only formed clear zones on R2A-PHBH agar plates, but also decreased the transparency of PHBH films and caused cracks and rough surfaces (Fig. 3B). These results demonstrated that the order *Burkholderiales* in biofilms functions as a degrader of PHBH films.

In conclusion, we herein suggested that biofilm formation on the surfaces of plastic films was important for their degradation in freshwater environments. A large biofilm rapidly formed on PHBH plastic films and contained PHBH-degrading *Burkholderiales* such as the genera *Acidovorax* and *Undibacterium*. The underlying mechanisms for the degradation of PHBH films by freshwater isolates currently remain unknown. The degradation of PHA films in aqueous solutions has generally been performed by the secreted PHA depolymerase, and enzymatic degradation occurred on the surface of PHA films, resulting in weight loss (4). Although the extracellular PHA depolymerase from the genus *Acidovorax* has been reported (15), a similar PHA-degrading enzyme from the genus *Undibacterium* has not yet been identified. Further studies on the identification of novel PHA-degrading enzymes and the mechanisms responsible for the degradation of PHBH by these bacteria may contribute to the dynamics of the carbon cycle of biodegradative plastics in freshwater environments being elucidated in more detail.

## Nucleotide sequence accession number

The 16S rRNA gene sequences from PHBH-degrading isolates have been deposited in the DDBJ/ENA/GenBank database under accession numbers LC361218 to LC361245. MiSeq sequence data has been deposited in the DDBJ Read Archive (DRA) under accession number DRA006558.

## Figures and Tables

**Fig. 1:**
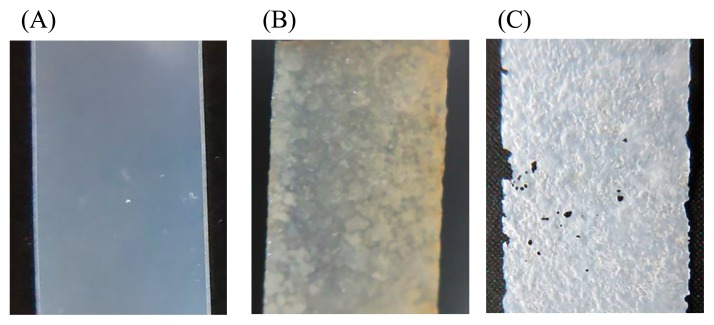
Biofilm formation on PHBH film surfaces in the freshwater sample obtained from Lake Yamanaka-ko. (A) Untreated PHBH film, (B) a biofilm formed on the PHBH film after an incubation for 2 weeks, and (C) a degraded PHBH film after the washout of the biofilm.

**Fig. 2:**
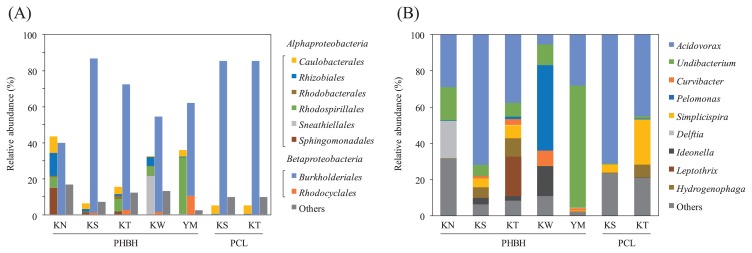
Microbiome analysis of biofilms formed on PHBH and PCL film surfaces. (A) Relative abundance of bacterial orders belonging to *Alphaproteobacteria* (left stacked bar), *Betaproteobacteria* (center stacked bar), or other classes (right stacked bar). (B) Relative abundance of bacterial genera for the order *Burkholderiales*.

**Fig. 3:**
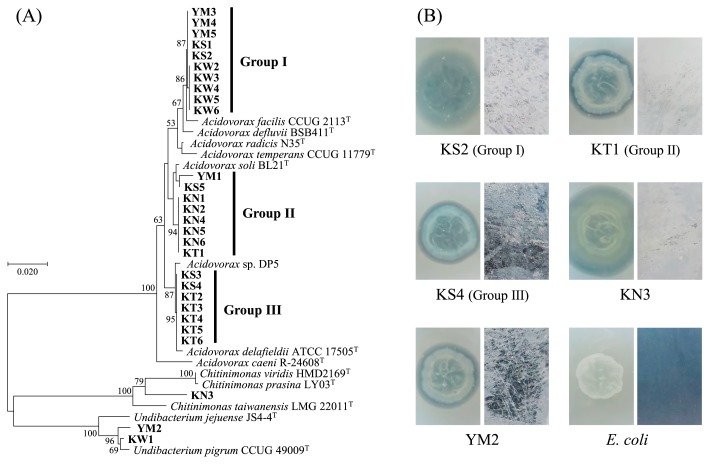
(A) Phylogenetic tree of 16S rRNA gene sequences from PHBH-degrading isolates. The bacterial isolates in the present study were shown in bold style. The phylogenetic tree was constructed by the neighbor-joining method with the ClustalW program of MEGA. Bootstrap values (500 resampling, ≥50%) are shown at the nodes. The scale bar represents 0.02 substitutions per nucleotide position. (B) PHBH-degrading activity of PHBH-degrading strains (KS2, KT1, KS4, KN3, and YM2) and a negative control strain (*E. coli*). Clear zones on R2A-PHBH agar plates (left) and the condition of the PHBH film surface (right) were observed after an incubation at 30°C for 3 d.

**Table 1:** Biofilm formation on plastic film surfaces in seawater samples

Sample name	KN	KS	KT	KW	YM
Sites	Kinugawa River	Lake Kasumigaura	Lake Kitaura	Lake Kawaguchi-ko	Lake Yamanaka-ko
Locate	36°54.15′ N 139°94.76′ E	36°09.61′ N 140°40.14′ E	36°11.25′ N 140°53.39′ E	35°50.43′ N 138°76.85′ E	35°40.82′ N 138°87.26′ E
Date	Mar 11	Apr 2	Apr 2	Apr 22	Apr 22
PLA	−	−	−	−	−
PBAT	+	+	+	+	+
PBS	+	++	+	+	+
PBSA	+	+	+	++	++
PCL	++	++	++	++	++
PHBH	++	++	++	++	++

a −, without a biofilm.

+, a biofilm is partially present, ++, the entire surface is covered by a biofilm
